# Poly[bis­[μ-1,4-bis­(imidazol-1-yl)butane]dicyanato­cadmium(II)]

**DOI:** 10.1107/S1600536809043104

**Published:** 2009-11-04

**Authors:** Xia Zhu, Ying Guo, Yun-Ling Zou

**Affiliations:** a Science College, Civil Aviation University of China, Tianjin 300300, People’s Republic of China

## Abstract

The coordination geometry of the Cd^II^ atom in the title complex, [Cd(NCO)_2_(C_10_H_14_N_4_)_2_]_*n*_ or [Cd(NCO)_2_(bimb)_2_]_*n*_, where bimb is 1,4-bis­(imidazol-1-yl)butane, is distorted octa­hedral with the Cd^II^ atom located on an inversion center and connected to four N atoms from the imidazole units of four symmetry-related bimb ligands and two O atoms from two symmetry-related NCO^−^ ligands. The Cd^II^ atoms are bridged by four bimb ligands, forming a two-dimensional (4,4) network.

## Related literature

For the synthesis and structure of 1,4-bis­(imidazol-1-yl)butane (bimb) complexes, see: Duncan *et al.* (1996 ▶); Ma *et al.* (2000 ▶); Yang *et al.* (2005 ▶); Zhang *et al.* (2008 ▶).
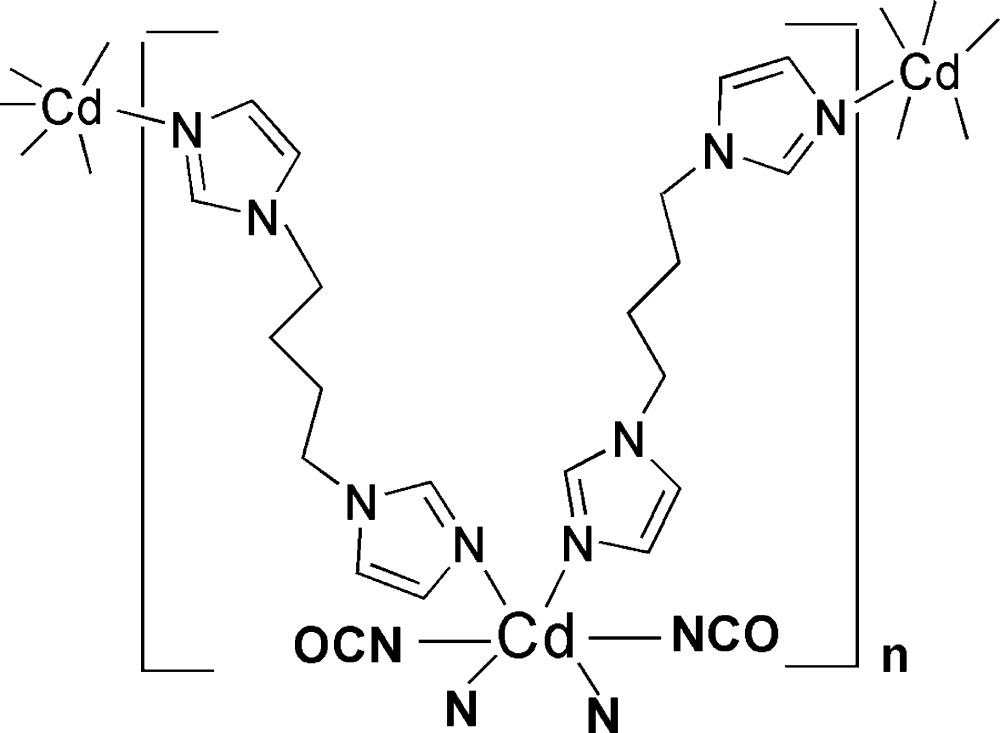


## Experimental

### 

#### Crystal data

[Cd(NCO)_2_(C_10_H_14_N_4_)_2_]
*M* *_r_* = 576.94
Monoclinic, 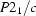

*a* = 7.7760 (14) Å
*b* = 18.156 (3) Å
*c* = 9.0983 (16) Å
β = 112.776 (3)°
*V* = 1184.4 (4) Å^3^
*Z* = 2
Mo *K*α radiation
μ = 0.96 mm^−1^
*T* = 153 K
0.45 × 0.35 × 0.30 mm

#### Data collection

Rigaku Mercury CCD diffractometer
Absorption correction: multi-scan (Jacobson, 1998 ▶) *T*_min_ = 0.671, *T*_max_ = 0.761
11270 measured reflections
2163 independent reflections
2066 reflections with *I* > 2σ(*I*)
*R*_int_ = 0.018

#### Refinement

*R*[*F* ^2^ > 2σ(*F* ^2^)] = 0.021
*wR*(*F* ^2^) = 0.056
*S* = 1.03
2163 reflections
161 parameters
H-atom parameters constrained
Δρ_max_ = 0.32 e Å^−3^
Δρ_min_ = −0.37 e Å^−3^

### 

Data collection: *CrystalClear* (Rigaku, 2000 ▶); cell refinement: *CrystalClear*; data reduction: *CrystalClear*; program(s) used to solve structure: *SHELXS97* (Sheldrick, 2008 ▶); program(s) used to refine structure: *SHELXL97* (Sheldrick, 2008 ▶); molecular graphics: *SHELXTL* (Sheldrick, 2008 ▶); software used to prepare material for publication: *SHELXTL*.

## Supplementary Material

Crystal structure: contains datablocks I, global. DOI: 10.1107/S1600536809043104/gk2236sup1.cif

Structure factors: contains datablocks I. DOI: 10.1107/S1600536809043104/gk2236Isup2.hkl

Additional supplementary materials:  crystallographic information; 3D view; checkCIF report

## Figures and Tables

**Table:** 

Cd1—N5	2.329 (2)
Cd1—N2	2.3276 (15)
Cd1—N4^i^	2.3800 (16)

**Table:** 

N5—Cd1—N2	88.27 (6)
N5—Cd1—N4^i^	87.83 (6)
N2—Cd1—N4^ii^	90.51 (5)

Symmetry codes: (i) 
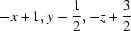
; (ii) 
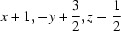
.

**Fig. 1.**
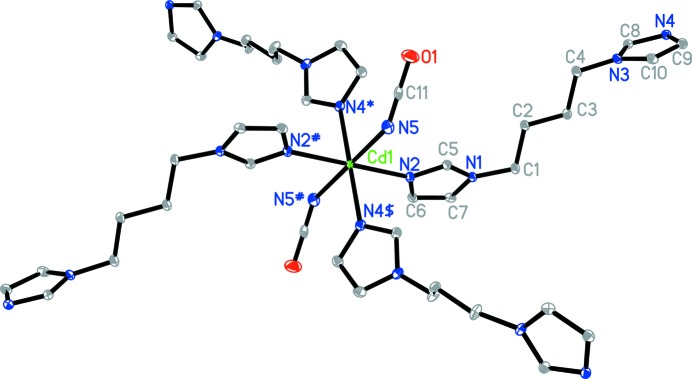
The coordination environment of the CoII atom in the title compound with the displacement ellipsoids at the 30% probability level. [Symmetry codes: # -x + 2, -y + 1, -z + 1; $ -x + 1, y - 1/2, -z + 3/2; * x + 1, -y + 3/2, z - 1/2]. Hydrogen atoms have been omitted for clarity.

**Fig. 2.**
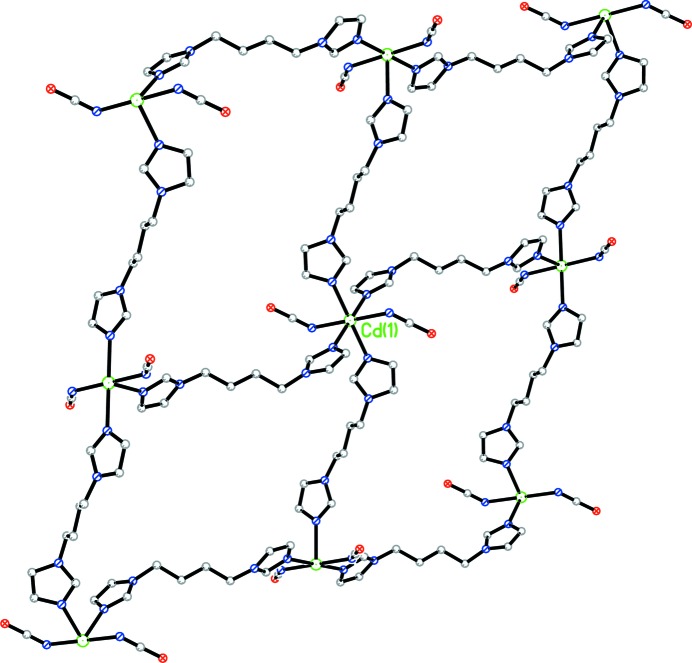
View of the two-dimensional (4,4) network of the title compound along the c direction.

**Fig. 3.**
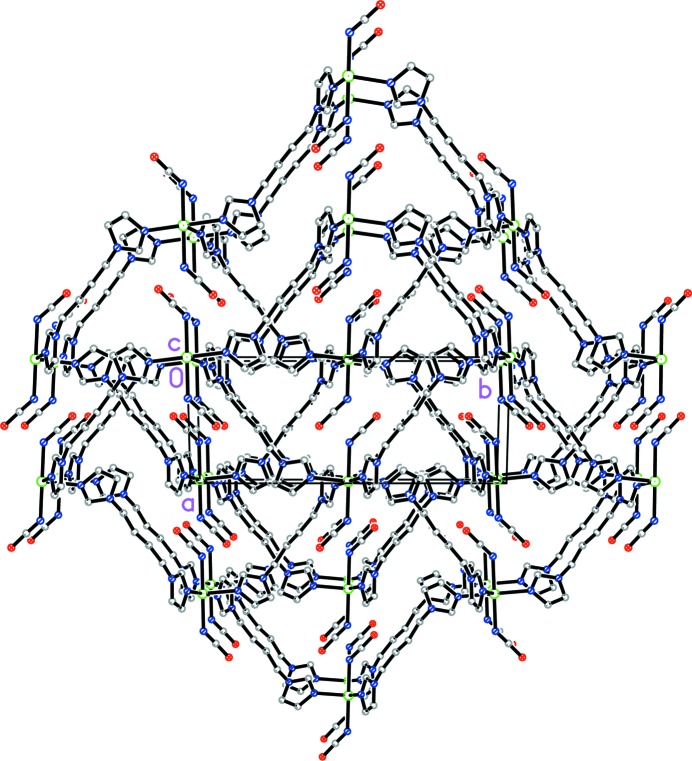
The cell packing of the title compound.

## supplementary crystallographic information

### Comment

The coordination environment of the Cd^II^ atom in the title compound is shown in Fig. 1. Each Cd^II^ atom is situated at the center of the symmetry. The coordination geometry of the Cd^II^ atom is distorted octahedral, with the metal center coordinated equatorially by four nitrogen atoms from four symmetry-related bimb ligands [Cd1—N2, 2.3276 (15) Å; Cd1—N4 2.3800 (16) Å], and axially by two nitrogen atoms from two cyanate anions [Cd1—N5 2.329 (2) Å]. Each bimb molecule exhibits the all-anti conformation of the tetramethylene linker. The torsion angles N1—C1—C2—C3, C1—C2—C3—C4 and C2—C3—C4—N3 are -166.64 (16), -173.74 (17) and 177.00 (16)°, respectively. The dihedral angle between the two imidazole rings in the ligand planes is 51.15 (8)°. Each Cd^II^ atom is bridged by four bimb ligands to form a neutral two-dimensional (4,4) network (Fig. 2). The networks contain square grids (44-membered ring), with a Cd^II^ atom at each corner and a bimb molecule at each edge connecting two Cd^II^ atoms. The edge lengths are 13.8184 (14) Å, which is obviously longer than the corresponding Cd^···^Cd separation (9.0819 (2) Å) for [Cd(bimb)_2_(NCS)_2_]_n_ in which bimb ligands show the *gauche*-anti-*gauche* conformation (Zhang *et al.*, 2008).

The two-dimensional networks parallel to (102) are stacked in an offset fashion along the c direction. In the superposition structure, the networks are arranged in the sequence ···A—B—A—B··· mode (Fig. 3). The cyanate anions are located in the voids.

[Cd(bimb)_2_(NCS)_2_]_n_ has an one-dimensional chain structure with double bridging bimb ligands (Zhang *et al.*, 2008). In the present work a two-dimensional cadmium(II) coordination polymer with the (4,4) network was synthesized when cyanate anions were used instead of thiocyanate anions. The factors which play the key role in the construction of the coordination polymers are not very clear. More work is need to extend the knowledge of the coordination polymers.

### Experimental

A 20 ml H_2_O/MeOH solution (1:1 *v*/*v*) of Cd(NO_3_)_2_^.^4H_2_O (0.154 g, 0.5 mmol) was added to one leg of an "H-shaped" tube, and a 20 ml H_2_O/MeOH (1:1 *v*/*v*) solution of bimb (0.190 g, 1.0 mmol) and NaNCO (0.065 g, 1.0 mmol) was added to the other leg of the tube. After two weeks, the well shaped colorless single crystals 1 were obtained. Yield: 64%. Found: C, 45.67; H, 4.82; N, 24.16. Calcd. for C_22_H_28_CdN_10_O_2_ (1): C, 45.80; H, 4.89; N, 24.28%.

### Refinement

H atom were placed in idealized positions and refined as riding, with C—H distances of 0.95 (imidazole) and 0.99Å (butane), and with *U*_iso_(H) = 1.2 times *U*_eq_(C).

### Crystal data

**Table:**

[Cd(NCO)_2_(C_10_H_14_N_4_)_2_]	*F*(000) = 588
*M**_r_* = 576.94	*D*_x_ = 1.618 Mg m^−^^3^
Monoclinic, *P*2_1_/*c*	Mo *K*α radiation, λ = 0.71070 Å
Hall symbol: -P 2ybc	Cell parameters from 4682 reflections
*a* = 7.7760 (14) Å	θ = 3.1–25.4°
*b* = 18.156 (3) Å	µ = 0.96 mm^−^^1^
*c* = 9.0983 (16) Å	*T* = 153 K
β = 112.776 (3)°	Block, colorless
*V* = 1184.4 (4) Å^3^	0.45 × 0.35 × 0.30 mm
*Z* = 2	

### Data collection

**Table:**

Rigaku Mercury CCD diffractometer	2163 independent reflections
Radiation source: fine-focus sealed tube	2066 reflections with *I* > 2σ(*I*)
graphite	*R*_int_ = 0.018
ω scans	θ_max_ = 25.3°, θ_min_ = 3.1°
Absorption correction: multi-scan (Jacobson, 1998)	*h* = −9→9
*T*_min_ = 0.671, *T*_max_ = 0.761	*k* = −21→19
11270 measured reflections	*l* = −9→10

### Refinement

**Table:**

Refinement on *F*^2^	Primary atom site location: structure-invariant direct methods
Least-squares matrix: full	Secondary atom site location: difference Fourier map
*R*[*F*^2^ > 2σ(*F*^2^)] = 0.021	Hydrogen site location: inferred from neighbouring sites
*wR*(*F*^2^) = 0.056	H-atom parameters constrained
*S* = 1.03	*w* = 1/[σ^2^(*F*_o_^2^) + (0.031*P*)^2^ + 0.9885*P*] where *P* = (*F*_o_^2^ + 2*F*_c_^2^)/3
2163 reflections	(Δ/σ)_max_ < 0.001
161 parameters	Δρ_max_ = 0.32 e Å^−^^3^
0 restraints	Δρ_min_ = −0.37 e Å^−^^3^

### Special details

**Table:**

Geometry. All e.s.d.'s (except the e.s.d. in the dihedral angle between two l.s. planes) are estimated using the full covariance matrix. The cell e.s.d.'s are taken into account individually in the estimation of e.s.d.'s in distances, angles and torsion angles; correlations between e.s.d.'s in cell parameters are only used when they are defined by crystal symmetry. An approximate (isotropic) treatment of cell e.s.d.'s is used for estimating e.s.d.'s involving l.s. planes.
Refinement. Refinement of *F*^2^ against ALL reflections. The weighted *R*-factor *wR* and goodness of fit *S* are based on *F*^2^, conventional *R*-factors *R* are based on *F*, with *F* set to zero for negative *F*^2^. The threshold expression of *F*^2^ > σ(*F*^2^) is used only for calculating *R*-factors(gt) *etc*. and is not relevant to the choice of reflections for refinement. *R*-factors based on *F*^2^ are statistically about twice as large as those based on *F*, and *R*- factors based on ALL data will be even larger.

### Fractional atomic coordinates and isotropic or equivalent isotropic displacement parameters (Å^2^)

**Table:**

	*x*	*y*	*z*	*U*_iso_*/*U*_eq_	
Cd1	1.0000	0.5000	0.5000	0.01448 (8)	
O1	0.4601 (2)	0.59262 (11)	0.2107 (2)	0.0527 (5)	
N1	0.8341 (2)	0.58976 (8)	0.88528 (18)	0.0175 (3)	
N2	0.9580 (2)	0.54469 (8)	0.72359 (18)	0.0176 (3)	
N3	0.1518 (2)	0.78895 (8)	0.82212 (18)	0.0192 (3)	
N4	0.0347 (2)	0.87774 (8)	0.92119 (18)	0.0185 (3)	
N5	0.6775 (3)	0.50356 (9)	0.3636 (2)	0.0261 (4)	
C1	0.6951 (3)	0.61731 (12)	0.9447 (2)	0.0247 (4)	
H1A	0.7592	0.6460	1.0435	0.030*	
H1B	0.6323	0.5750	0.9715	0.030*	
C2	0.5502 (3)	0.66549 (10)	0.8233 (2)	0.0191 (4)	
H2A	0.4667	0.6342	0.7356	0.023*	
H2B	0.6135	0.7005	0.7775	0.023*	
C3	0.4331 (3)	0.70868 (10)	0.8957 (2)	0.0188 (4)	
H3A	0.3794	0.6745	0.9514	0.023*	
H3B	0.5131	0.7446	0.9746	0.023*	
C4	0.2777 (3)	0.74894 (12)	0.7648 (2)	0.0255 (4)	
H4A	0.3335	0.7843	0.7130	0.031*	
H4B	0.2042	0.7128	0.6831	0.031*	
C5	0.8009 (3)	0.56271 (10)	0.7382 (2)	0.0182 (4)	
H5A	0.6804	0.5574	0.6562	0.022*	
C6	1.0980 (3)	0.56036 (10)	0.8684 (2)	0.0189 (4)	
H6A	1.2273	0.5528	0.8940	0.023*	
C7	1.0241 (3)	0.58814 (10)	0.9689 (2)	0.0201 (4)	
H7A	1.0902	0.6035	1.0759	0.024*	
C8	0.1767 (3)	0.85724 (10)	0.8854 (2)	0.0187 (4)	
H8A	0.2833	0.8869	0.9021	0.022*	
C9	−0.0872 (3)	0.81930 (10)	0.8791 (2)	0.0214 (4)	
H9A	−0.2033	0.8177	0.8910	0.026*	
C10	−0.0166 (3)	0.76412 (11)	0.8180 (2)	0.0227 (4)	
H10A	−0.0727	0.7177	0.7801	0.027*	
C11	0.5754 (3)	0.54682 (11)	0.2911 (2)	0.0210 (4)	

### Atomic displacement parameters (Å^2^)

**Table:**

	*U*^11^	*U*^22^	*U*^33^	*U*^12^	*U*^13^	*U*^23^
Cd1	0.01502 (12)	0.01346 (12)	0.01762 (12)	−0.00031 (6)	0.00926 (9)	−0.00038 (6)
O1	0.0318 (9)	0.0588 (12)	0.0659 (13)	0.0157 (9)	0.0171 (9)	0.0309 (10)
N1	0.0191 (8)	0.0189 (8)	0.0164 (7)	0.0038 (6)	0.0088 (6)	0.0004 (6)
N2	0.0176 (8)	0.0172 (8)	0.0205 (8)	−0.0001 (6)	0.0101 (6)	−0.0011 (6)
N3	0.0209 (8)	0.0190 (8)	0.0181 (8)	0.0066 (6)	0.0081 (7)	0.0010 (6)
N4	0.0203 (8)	0.0170 (8)	0.0195 (8)	0.0025 (6)	0.0092 (6)	0.0015 (6)
N5	0.0176 (9)	0.0289 (11)	0.0310 (11)	−0.0048 (7)	0.0084 (8)	−0.0031 (7)
C1	0.0272 (10)	0.0315 (11)	0.0202 (10)	0.0113 (8)	0.0144 (8)	0.0028 (8)
C2	0.0199 (9)	0.0192 (9)	0.0199 (9)	0.0027 (7)	0.0097 (8)	−0.0016 (7)
C3	0.0199 (9)	0.0188 (9)	0.0192 (9)	0.0016 (7)	0.0094 (8)	−0.0025 (7)
C4	0.0300 (11)	0.0276 (10)	0.0221 (9)	0.0113 (9)	0.0136 (9)	0.0003 (8)
C5	0.0181 (9)	0.0194 (9)	0.0171 (9)	0.0011 (7)	0.0068 (7)	0.0002 (7)
C6	0.0149 (9)	0.0180 (9)	0.0225 (9)	0.0010 (7)	0.0060 (7)	0.0016 (7)
C7	0.0198 (9)	0.0179 (10)	0.0194 (9)	0.0016 (7)	0.0039 (8)	0.0003 (8)
C8	0.0199 (9)	0.0191 (9)	0.0179 (9)	0.0023 (7)	0.0081 (8)	0.0023 (7)
C9	0.0200 (9)	0.0186 (9)	0.0260 (10)	0.0006 (7)	0.0092 (8)	0.0019 (8)
C10	0.0238 (10)	0.0154 (9)	0.0267 (10)	0.0011 (7)	0.0073 (8)	0.0010 (8)
C11	0.0159 (9)	0.0280 (11)	0.0235 (10)	−0.0061 (9)	0.0122 (8)	−0.0065 (9)

### Geometric parameters (Å, °)

**Table:**

Cd1—N5	2.329 (2)	C1—C2	1.514 (3)
Cd1—N5^i^	2.329 (2)	C1—H1A	0.9900
Cd1—N2	2.3276 (15)	C1—H1B	0.9900
Cd1—N2^i^	2.3276 (15)	C2—C3	1.530 (2)
Cd1—N4^ii^	2.3800 (16)	C2—H2A	0.9900
Cd1—N4^iii^	2.3800 (16)	C2—H2B	0.9900
O1—C11	1.234 (3)	C3—C4	1.516 (3)
N1—C5	1.353 (2)	C3—H3A	0.9900
N1—C7	1.375 (2)	C3—H3B	0.9900
N1—C1	1.471 (2)	C4—H4A	0.9900
N2—C5	1.320 (2)	C4—H4B	0.9900
N2—C6	1.375 (2)	C5—H5A	0.9500
N3—C8	1.349 (2)	C6—C7	1.352 (3)
N3—C10	1.372 (3)	C6—H6A	0.9500
N3—C4	1.468 (2)	C7—H7A	0.9500
N4—C8	1.320 (2)	C8—H8A	0.9500
N4—C9	1.375 (2)	C9—C10	1.360 (3)
N4—Cd1^iv^	2.3800 (16)	C9—H9A	0.9500
N5—C11	1.130 (3)	C10—H10A	0.9500
			
N5—Cd1—N5^i^	180.0	C3—C2—H2A	109.1
N5—Cd1—N2	88.27 (6)	C1—C2—H2B	109.1
N5^i^—Cd1—N2	91.73 (6)	C3—C2—H2B	109.1
N5—Cd1—N2^i^	91.73 (6)	H2A—C2—H2B	107.9
N5^i^—Cd1—N2^i^	88.27 (6)	C4—C3—C2	109.55 (15)
N2—Cd1—N2^i^	180.000 (1)	C4—C3—H3A	109.8
N5—Cd1—N4^ii^	87.83 (6)	C2—C3—H3A	109.8
N5^i^—Cd1—N4^ii^	92.17 (6)	C4—C3—H3B	109.8
N2—Cd1—N4^ii^	89.49 (5)	C2—C3—H3B	109.8
N2^i^—Cd1—N4^ii^	90.51 (5)	H3A—C3—H3B	108.2
N5—Cd1—N4^iii^	92.17 (6)	N3—C4—C3	113.43 (16)
N5^i^—Cd1—N4^iii^	87.83 (6)	N3—C4—H4A	108.9
N2—Cd1—N4^iii^	90.51 (5)	C3—C4—H4A	108.9
N2^i^—Cd1—N4^iii^	89.49 (5)	N3—C4—H4B	108.9
N4^ii^—Cd1—N4^iii^	180.0	C3—C4—H4B	108.9
C5—N1—C7	107.00 (16)	H4A—C4—H4B	107.7
C5—N1—C1	126.94 (16)	N2—C5—N1	111.08 (16)
C7—N1—C1	126.05 (16)	N2—C5—H5A	124.5
C5—N2—C6	105.81 (15)	N1—C5—H5A	124.5
C5—N2—Cd1	128.51 (12)	N2—C6—C7	109.82 (16)
C6—N2—Cd1	125.64 (12)	N2—C6—H6A	125.1
C8—N3—C10	106.97 (16)	C7—C6—H6A	125.1
C8—N3—C4	126.95 (17)	C6—C7—N1	106.29 (17)
C10—N3—C4	126.07 (17)	C6—C7—H7A	126.9
C8—N4—C9	105.44 (16)	N1—C7—H7A	126.9
C8—N4—Cd1^iv^	122.73 (12)	N4—C8—N3	111.63 (17)
C9—N4—Cd1^iv^	131.09 (12)	N4—C8—H8A	124.2
C11—N5—Cd1	134.36 (15)	N3—C8—H8A	124.2
N1—C1—C2	111.94 (15)	C10—C9—N4	109.78 (17)
N1—C1—H1A	109.2	C10—C9—H9A	125.1
C2—C1—H1A	109.2	N4—C9—H9A	125.1
N1—C1—H1B	109.2	C9—C10—N3	106.18 (17)
C2—C1—H1B	109.2	C9—C10—H10A	126.9
H1A—C1—H1B	107.9	N3—C10—H10A	126.9
C1—C2—C3	112.39 (15)	N5—C11—O1	178.3 (2)
C1—C2—H2A	109.1		
			
N5—Cd1—N2—C5	−1.34 (16)	C6—N2—C5—N1	0.5 (2)
N5^i^—Cd1—N2—C5	178.66 (16)	Cd1—N2—C5—N1	−177.22 (11)
N4^ii^—Cd1—N2—C5	−89.19 (16)	C7—N1—C5—N2	−0.5 (2)
N4^iii^—Cd1—N2—C5	90.81 (16)	C1—N1—C5—N2	178.33 (17)
N5—Cd1—N2—C6	−178.67 (15)	C5—N2—C6—C7	−0.4 (2)
N5^i^—Cd1—N2—C6	1.33 (15)	Cd1—N2—C6—C7	177.45 (12)
N4^ii^—Cd1—N2—C6	93.49 (15)	N2—C6—C7—N1	0.1 (2)
N4^iii^—Cd1—N2—C6	−86.51 (15)	C5—N1—C7—C6	0.2 (2)
N2—Cd1—N5—C11	88.7 (2)	C1—N1—C7—C6	−178.60 (17)
N2^i^—Cd1—N5—C11	−91.3 (2)	C9—N4—C8—N3	0.2 (2)
N4^ii^—Cd1—N5—C11	178.2 (2)	Cd1^iv^—N4—C8—N3	−170.90 (11)
N4^iii^—Cd1—N5—C11	−1.8 (2)	C10—N3—C8—N4	−0.3 (2)
C5—N1—C1—C2	−43.3 (3)	C4—N3—C8—N4	178.41 (17)
C7—N1—C1—C2	135.28 (19)	C8—N4—C9—C10	−0.1 (2)
N1—C1—C2—C3	−166.64 (16)	Cd1^iv^—N4—C9—C10	170.02 (13)
C1—C2—C3—C4	−173.74 (17)	N4—C9—C10—N3	−0.1 (2)
C8—N3—C4—C3	84.5 (2)	C8—N3—C10—C9	0.2 (2)
C10—N3—C4—C3	−97.0 (2)	C4—N3—C10—C9	−178.49 (17)
C2—C3—C4—N3	177.00 (16)		

Symmetry codes: (i) −*x*+2, −*y*+1, −*z*+1; (ii) −*x*+1, *y*−1/2, −*z*+3/2; (iii) *x*+1, −*y*+3/2, *z*−1/2; (iv) −*x*+1, *y*+1/2, −*z*+3/2.
